# Supplementary material for: NYUS.2: an automated machine learning prediction model for the large-scale real-time simulation of grapevine freezing tolerance in North America
Source: Hortic Res. 2023 Dec 29;11(2):uhad286. doi: 10.1093/hr/uhad286 (PMC10939402; doi:10.1093/hr/uhad286)
Supplement: Web_Material_uhad286 [file web_material_uhad286.zip › Wang_etal_SI_Table_Figure_Note_HortRe_edited.docx]

## Supplemental Materials

Article title: NYUS.2: an Automated Machine Learning Prediction Model for the Large-scale Real-time Simulation of Grapevine Freezing Tolerance in North America

Authors: Hongrui Wang, Gaurav D. Moghe, Al P. Kovaleski, Markus Keller, Timothy E. Martinson, A. Harrison Wright, Jeffrey L. Franklin, Andréanne Hébert-Haché, Caroline Provost, Michael Reinke, Amaya Atucha, Michael G. North, Jennifer P. Russo, Pierre Helwi, Michela Centinari and Jason P. Londo

**Table S1** **Data collection site detail**

**Table S2** **Technical term definition**

**Figure S1 Result of the method suitability test and the site-transferability test**

**Figure S2 Maximum potential that freezing damage has occurred in 16 grapevine cultivars in the 2022-2023 dormant season**

**Figure S3 Maximum freezing tolerance of 16 grapevine cultivars in the 2022-2023 dormant season**

**Figure S4 Impact of different feature groups on Auto-ML model prediction**

**Figure S5 Auto-ML model utilization of three chilling models**

**Figure S6** **Correlation analysis of EWMA and REWMA of daily minimum temperature**

**Note S1** **Limitations of mechanistic modeling and DL-empowered modeling in plant physiology**

**Note S2** **Feature importance investigation based on feature groups**

**Note S3** **Analysis of the utilization of three chilling models in the Auto-ML model**

**Note S4** **Daily temperature descriptors and cumulative temperature descriptors**

**Note S5** **Reverse exponential weighted moving average temperatures in the modeling of grapevine freezing tolerance**

The following supplemental materials are available for this article:

**Table S1 Data collection site detail**

|  | **Site detail** | | | | **Cultivar detail** | | |
| --- | --- | --- | --- | --- | --- | --- | --- |
| Location | longitude | latitude | Weather data | Station ID | Cultivar number | Record | Cultivars |
| BC | -119.60 | 49.46 | DACCD*^1^* | Penticton A | 15 | 2012-11-01 to 2022-04-07 | Cabernet Franc, Cabernet Sauvignon, Chardonnay, Gewurztraminer, Malbec, Merlot, Pinot blanc, Pinot gris, Pinot noir, Riesling, Sauvignon blanc, Syrah, Tempranillo, Viognier, Zinfandel |
| NY | -77.03 | 42.88 | NEWA*^2^* | Bejo | 32 | 2012-10-31 to 2022-03-07 | Aromella, Cabernet Franc, Cabernet Sauvignon, Cayuga White, Chambourcin, Chancellor, Chardonnay, Chenin blanc, Concord, Corot noir, Gewurztraminer, Gruner Veltliner, La Crescent, Lemberger, Marechal Foch, Marquette, Merlot, Niagara, Noiret, Pinot gris, Pinot noir, Riesling, Sangiovese, Saperavi, Sauvignon blanc, St. Croix, Syrah, Tocai Fruliano, Traminette, Valvin Muscat, Vidal, Vignoles |
| NS | -64.32 | 44.93 | On-site measurement*^3^* | - | 5 | 2018-10-29 to 2023-02-22 | Chardonnay, Marquette, Pinot noir, Riesling, L'Acadie |
| PA | -77.95 | 40.71 | NEWA | Rock Springs & Lewisburg (FERO vineyards) | 4 | 2018-11-06 to 2022-11-29 | Lemberger, Marquette, Noiret, Riesling |
| WA | -119.74 | 46.25 | Agweather | Prosser. NE & Paterson. E | 12 | 2005-09-28 to 2012-04-17 | Cabernet Franc, Cabernet Sauvignon, Chardonnay, Chenin blanc, Gewurztraminer, Lemberger, Merlot, Pinot gris, Riesling, Sangiovese, Sauvignon blanc, Syrah |
| QC | -74.05 | 45.49 | On-site measurement | - | 13 | 2020-10-20 to 2022-12-13 | Cabernet Franc, Chardonnay, Marquette, Pinot gris, Pinot noir, Riesling, Vidal, Frontenac, Petite Pearl, Frontenac blanc, Frontenac gris, Seyval, St. Pepin |
| MI | -86.36 | 42.08 | Enviroweather | SWMREC | 11 | 2021-11-23 to 2022-04-27 | Cabernet Franc, Cabernet Sauvignon, Concord, Marechal Foch, Marquette, Merlot, Niagara, Pinot gris, Pinot noir, Sauvignon blanc, Traminette |
| TX | -101.82 | 33.65 | ACIS*^4^* | USW00023042 | 4 | 2021-12-08 to 2022-02-16 | Cabernet Sauvignon, Sangiovese, Tempranillo, Viognier |
| WI | -89.53 | 43.06 | ACIS | USW00014837 | 5 | 2017-11-02 to 2020-04-17 | La Crescent, Marquette, Brianna, Frontenac, Petite Pearl |
| *^1^*Digital Archive of Canadian Climatological Data (DACCD) | | | | | | | |
| *^2^*Network for Environment and Weather Applications (NEWA) | | | | | | | |
| *^3^*Weather data was obtained from on-site weather stations | | | | | | | |
| *^4^*Applied Climate Information System (ACIS) | | | | | | | |

**Table S2 Technical term definition**

| **Term** | **Abbreviation** | **Definition** |
| --- | --- | --- |
| AutoGluon | **-** | An open-source automated machine learning toolkit from AWS designed to simplify and accelerate the process of training and optimizing machine learning models, with minimal input required from the user. The training engine of NYUS.2 model |
| Automated machine learning | **Auto-ML** | A process that automates the end-to-end process of applying machine learning to real-world problems, from data preprocessing to model selection and tuning. |
| Boolean-type feature | **-** | A binary variable in a dataset that represents information using only two distinct values, typically 0 and 1. |
| Cross validation | **-** | A statistical technique that partitions a dataset into subsets, training a model on one subset and validating it on another, to assess the model's performance and reduce overfitting |
| Deep learning | **DL** | A subset of machine learning that utilizes neural networks with many layers to analyze various forms of data, enabling advanced pattern recognition and decision-making. |
| Differential thermal analysis | **DTA** | A technique to measure LTE through continuous lowering temperature and recording heat release |
| Exponential weighted moving average | **EWMA** | A calculation that gives more weight to recent data points when computing moving average. |
| External testing | **-** | In this study, external testing refers to the testing of the model with the data that was neither used for training nor from the same locations of the training data. |
| Growing degree hours | **GDH** | A measure of heat accumulation used in agriculture to predict plant growth, calculated by integrating temperature over time within a specific range optimal. |
| Internal testing | **-** | In this study, internal testing refers to the testing of the model with the data that was not used for training but from the same locations of the training data. |
| Lethal temperature for 50% population | **LT_50_** | A measure of grapevine bud freezing tolerance |
| Low temperature exotherm | **LTE** | A burst of heat released when intracellular ice formation occurs |
| Machine learning | **ML** | A subset of artificial intelligence that enables computers to improve their performance on tasks by learning from data. |
| Recurrent neural network | **RNN** | A type of neural network designed to recognize patterns in sequences of data by looping its output back into its input, allowing it to maintain a form of memory. |
| Reverse exponential weighted moving average | **REWMA** | A calculation developed in this study that reversely compute EWMA by giving more weight to earlier data. |
| Root-mean-square error | **RMSE** | A metric that quantifies the difference between predicted and observed values, representing the square root of the average squared differences between them. |
| SHapley Additive exPlanations | **SHAP** | A method to interpret machine learning models by attributing the change in prediction outcome to each individual feature, based on cooperative game theory. |
| Weighted ensemble model | **-** | A weighted ensemble model combines multiple models' predictions, assigning different importance or weights to each, to achieve improved predictive performance over individual models. |


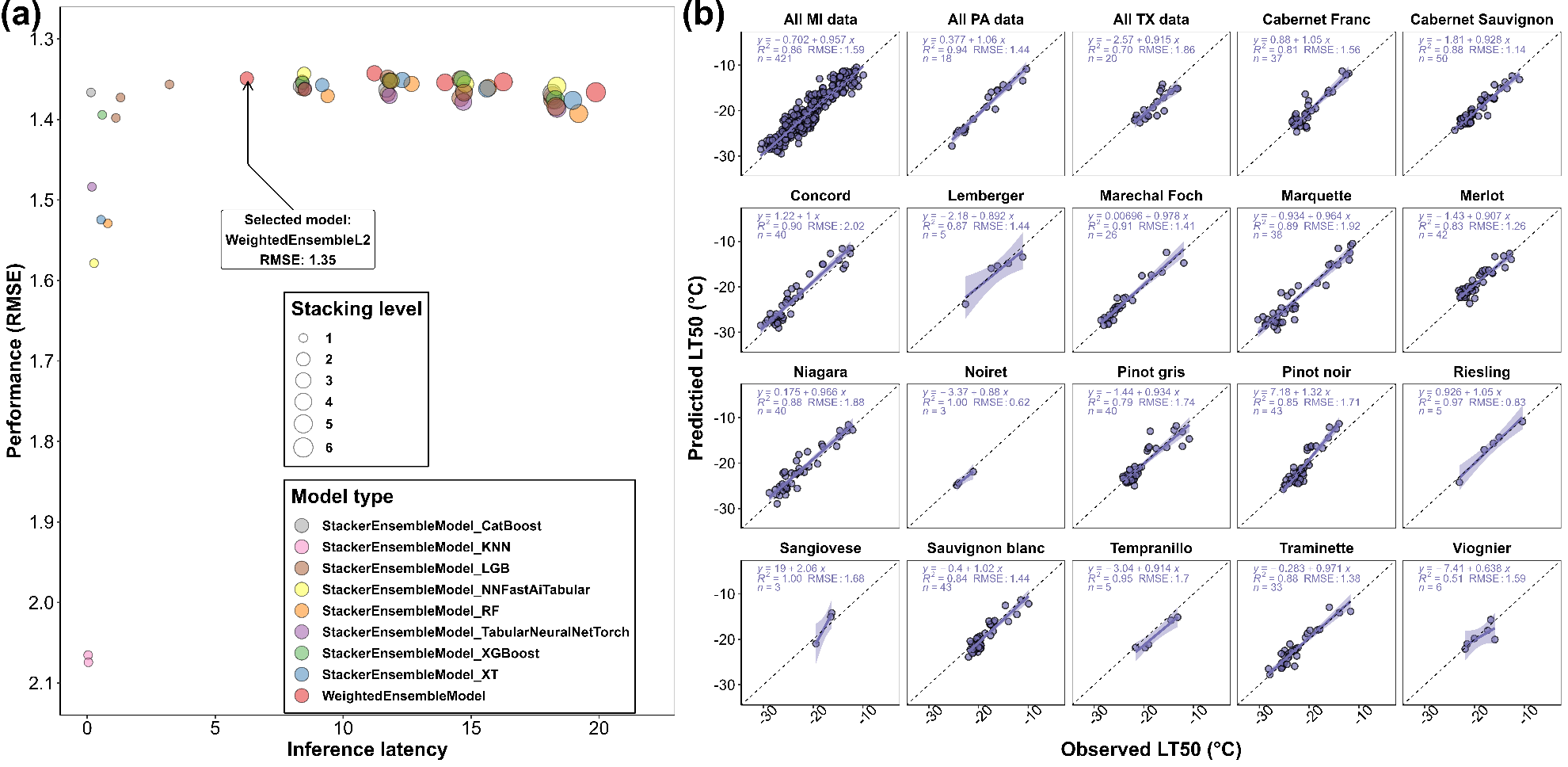
**Figure S1** Result of the method suitability test and the site-transferability test. (a) The performance (RMSE) of all the models generated from training on internal testing data ($n=970$). Inference latency refers the relative time it takes for a machine learning model to process and analyze data and produce an output. (b) The performance of the alpha grapevine LT_50_ prediction model on external testing data ($n=459$) by individual sub-datasets and cultivars.

**Figure S2** Maximum potential that freezing damage has occurred in 16 grapevine cultivars in the 2022-2023 dormant season. The potential that a freezing damage has occurred (0 to 100%), is estimated through a symmetric sigmoid function assuming that 10% and 90% of potential that a freezing damage has occurred when the ambient temperature is 2 °C above and below the predicted LT_50_, respectively


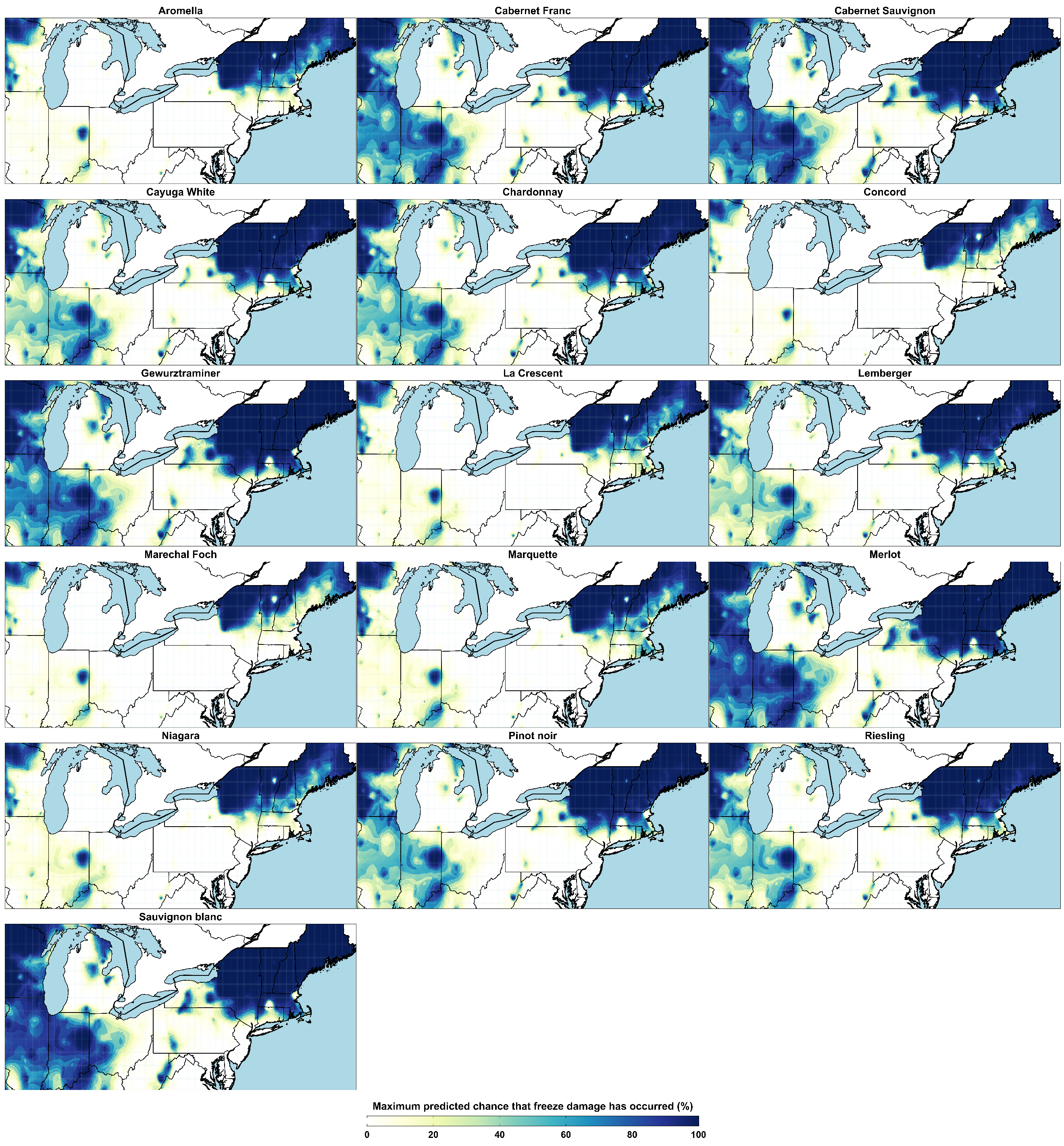


**Figure S3** Maximum freezing tolerance of 16 grapevine cultivars in the 2022-2023 dormant season. The freezing tolerance is expressed as LT_50_ (°C).


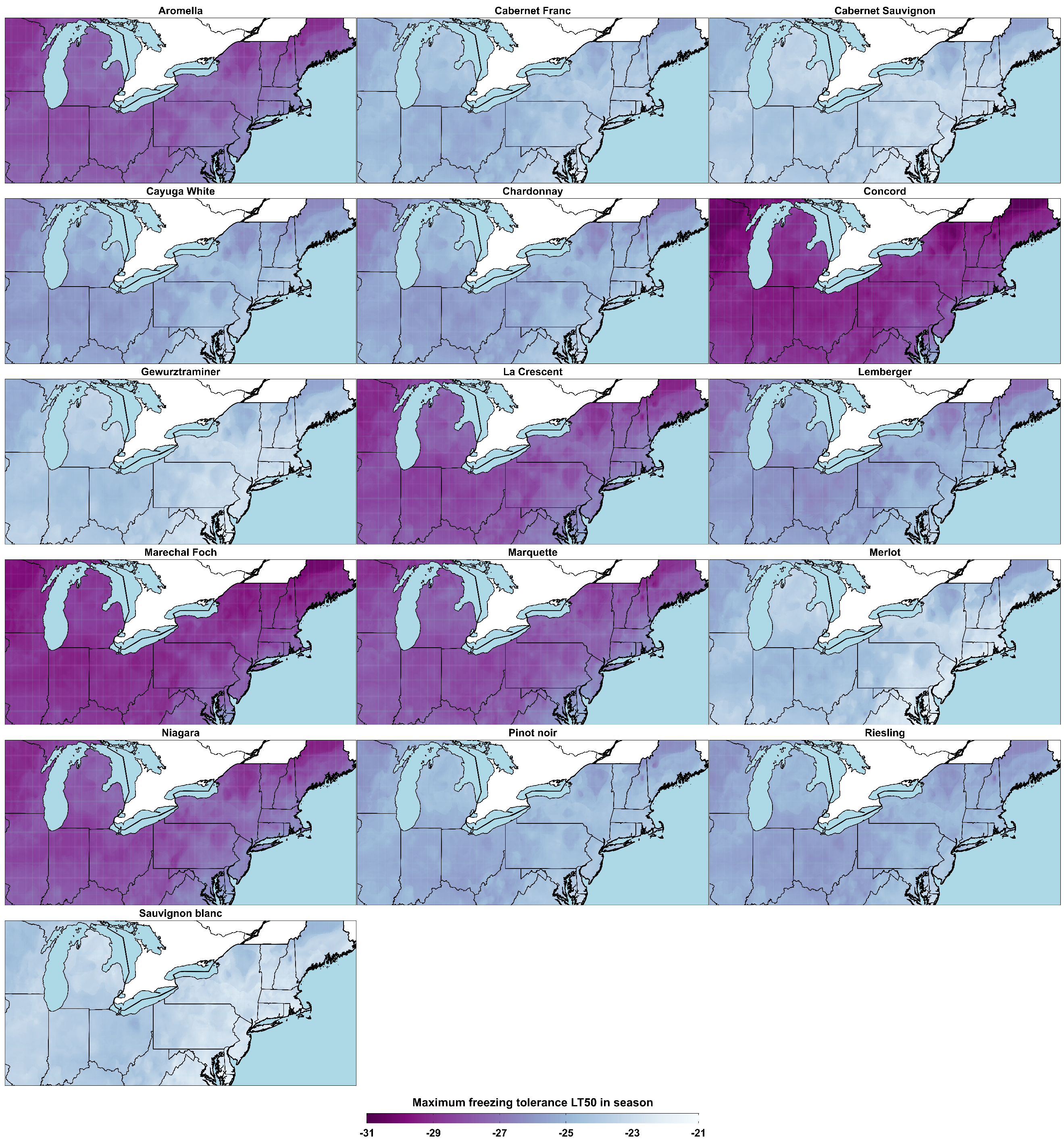


**Figure S4** Impact of different feature groups on Auto-ML model prediction. (a) Impact of top features on model prediction. The top features include the five shared features among the top 15 important features quantified by AutoGluon and SHAP value. (b) Impact of EWMA and REWMA temperatures on model prediction. EWMA and REWMA temperatures include 60 features computed with different moving average methods using daily temperatures. (c) Impact of cultivar features on model prediction. Cultivar features include 45 one-hot encoded cultivars. Internal testing data $(n=1,016)$ was used for the model prediction comparison. The plots are showing the prediction of the LT_50_ in Geneva, NY in the 2022-2023 dormant season.


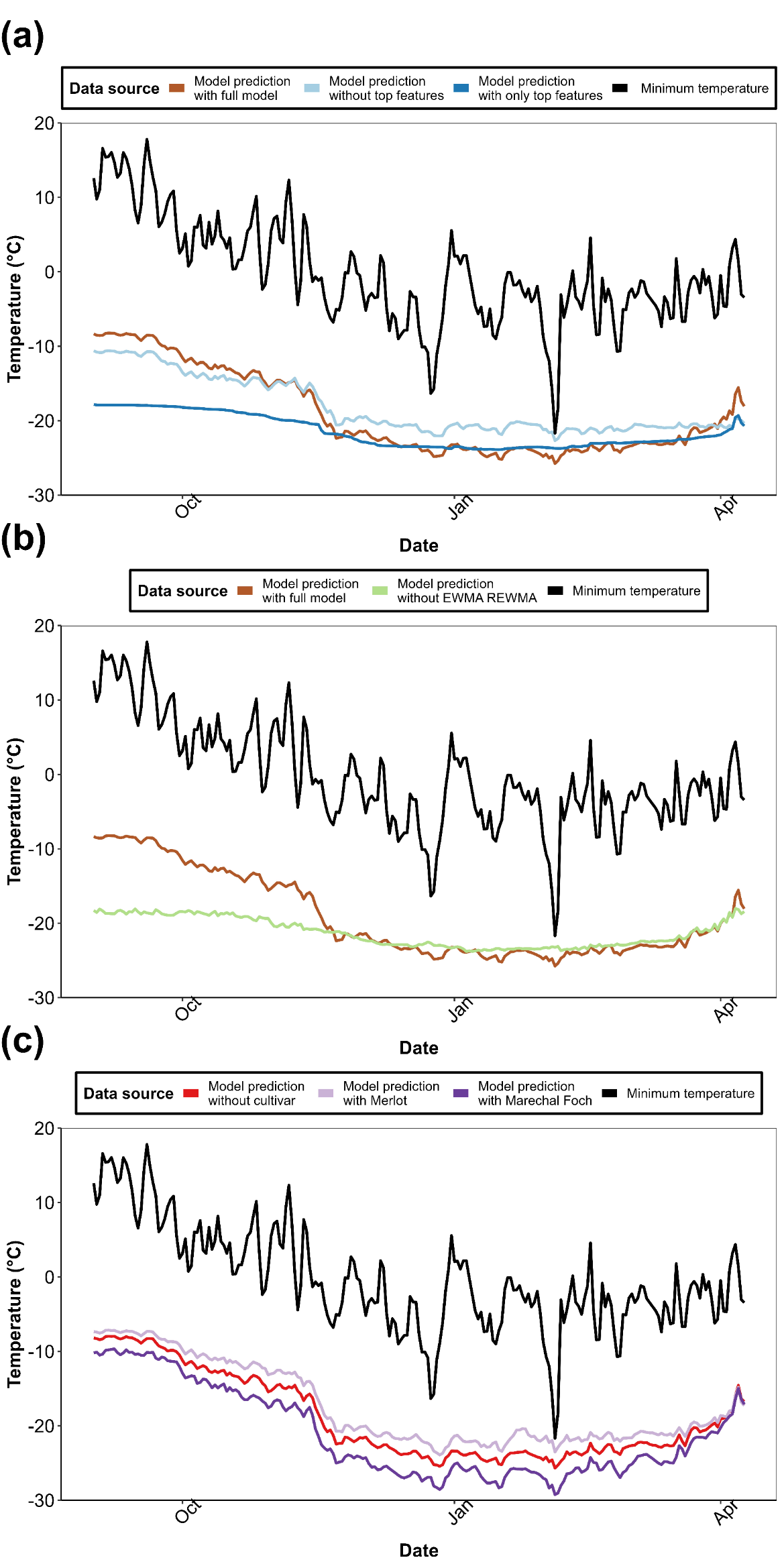


**Figure S5** Auto-ML model utilization of three chilling models. (a) The change of SHAP value with the increase of normalized feature values in three chilling models. Normalized feature value is calculated with $x_{normalized}=(x-x_{\min})/(x_{\max}-x_{\min})$ and ranged from 0 to 1. (b) The change of combined SHAP value with the increase of combined normalized feature value. Combined SHAP value is calculated by summing the SHAP value of the three chilling models. Combined normalized feature value is calculated by average the normalized feature values of the three chilling models. The trendline is fitted using ‘loess’ with $\alpha=0.7$. (c) Derivative of the fitted trendline over combined normalized feature value.


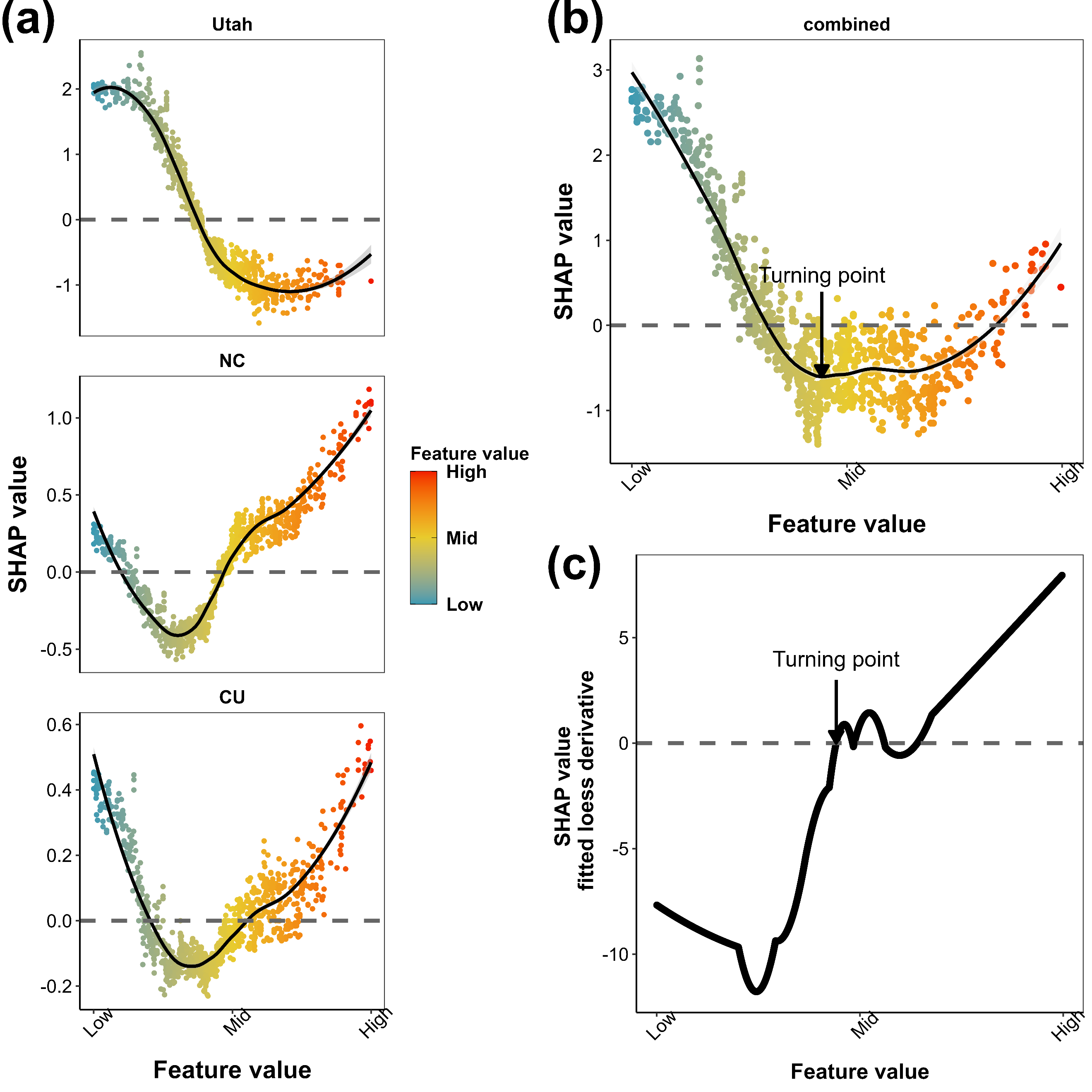


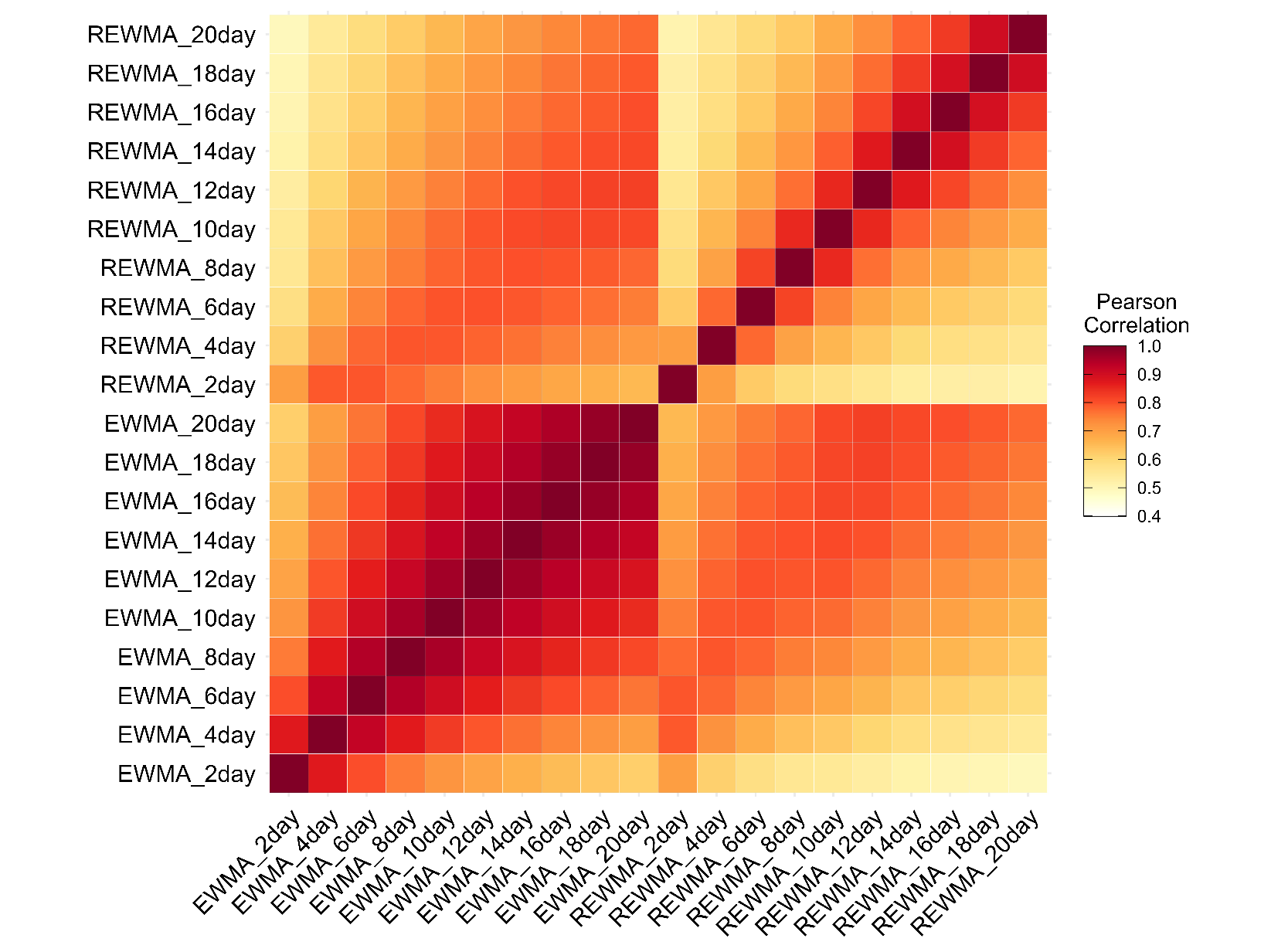
**Figure S6** Correlation analysis of EWMA and REWMA of daily minimum temperature. All the EWMA and REWMA of daily minimum temperatures in the entire dataset was used for the correlation analysis.

**Note S1** Limitations of mechanistic modeling and DL-empowered modeling in plant physiology

The leading issues with modeling plant physiology are usually the availability of training data and the ease of modeling itself. The WAUS.2 model is a product of mechanistic modeling that mathematically realizes an understanding of grapevine dormant season biology [1]. The NYUS.1 model uses a combination of mechanistic and empirical modeling through the formulation of empirically determined concepts of grapevine dormant season physiology [2]. Smaller training datasets are sufficient for training and generating such models. However, the models trained using such datasets tend to overfit niche conditions. In contrast, the RNN grapevine LT_50_ prediction model is a product of DL, in which unstructured weather data in the whole season are used for training [3]. However, generally, tens of thousands of training data are required to generate a stably functional model for plant physiology [4–6]. Thus, a potential challenge of this model is the potential underperformance due to the relatively low quantity of training data ($n< 8,000$). The RNN model cannot outperform WAUS.2 in some cultivars under Washington state weather conditions [3].

Mechanistic models and DL-empowered models are complicated to build, but the underlying reasons are different. Current constraints on traditional mechanistic modeling methods include the need for extensive and manual inputs and training requirements for both developers and end-users. For developers, model construction involves a thorough understanding of the biological systems for parameter selection and combination, which often results in complicated models that require specialized expertise to interpret [7]. For users, these issues can make mechanistic models less user-friendly and less likely to be adopted [8]. Regarding DL-empowered modeling, although the modeling itself involves less biological reasoning, the model selection, the design of the neural networks backbone, and the hyperparameter selection require intensive manual tuning by computer scientists and specialized computational hardware (e.g., GPUs), thus reducing adoption in the field of plant physiology [9].

**Note S2** Feature importance investigation based on feature groups

As we noticed that several most important features from feature importance quantification exhibited much higher importance scores than the other features, we precisely analyzed if the model only relies on these top features for prediction. The impact of two other groups of features, EWMA and REWMA temperature features and cultivar features, were also analyzed. Internal testing data $n=1,016$ were used for model prediction and performance comparisons between the full model and the models without certain feature groups.

The top features selected for the analysis are the five shared features from top 15 most important feature lists generated by AutoGluon and SHAP value. These features are the chilling accumulation estimation of Utah model, days in season, the EWMA of mean temperature with window size of 12, the chilling accumulation estimation of NC model, and the GDH with 10 °C as base temperature. By excluding these top features, the $RMSE$ of the model prediction on internal testing data increased from 1.36 °C (full model prediction) to 3.01 °C. By including only these top features, the $RMSE$ of the model prediction on internal testing data increased to 4.12 °C. An example of model prediction difference between the models without/with only the top features and the full model is shown in Figure S4a. Compared to the prediction from the full model, the prediction without the top features followed the same trend during cold acclimation in early season and the maintenance of maximum freezing tolerance in mid season but did not reveal the response of deacclimation in late season (Figure S4a). Compared to the prediction from the full model, the prediction with only these top features neither responded to daily temperature variations nor exhibited accuracy in early season (Figure S4a). These results indicate that, the top features are essential for the prediction of LT50 during deacclimation, however, the model does not only rely on these features for prediction.

The analysis of EWMA and REWMA temperature features incorporated the comparison between the performance of the model without 60 EWMA and REWMA temperature features and the full model. By excluding the EWMA and REWMA features, the $RMSE$ of the model prediction on internal testing data increased to 4.12 °C. The model without the EWMA and REWMA features lost prediction accuracy during cold acclimation in early season and did not reveal proper response to daily temperature changes (Figure S4b). The analysis of cultivar features incorporated the comparison between the performance of the model without 45 one-hot encoded cultivar features and the full model with proper cultivar features. The impact of using proper cultivar feature on model prediction is shown in Figure S4c, using the cultivars ‘Marechal Foch’ and ‘Merlot’ as examples. By excluding all the cultivar features, the model could not generate cultivar-specific prediction, and the predicted LT_50_s are most likely intermediate LT_50_s generalized among all the cultivars, thus might be used as a backbone to generate cultivar-specific prediction (Figure S4c). To compare, when adding proper cultivar features to the model, the predicted LT_50_s are calibrated to reveal cultivar-specific responses to temperature (Figure S4c). For example, ‘Marechal Foch’ tends to acclimate faster in early season, maintain lower LT_50_ in mid season and deacclimates faster in late season, as compared to ‘Merlot’ (Figure S4c). These results align with previous findings regarding the differentiation of acclimation and deacclimation dynamics in different grapevine cultivars, indicating that the cultivar-specific responses in freezing tolerance are addressed by the Auto-ML model with the cultivar features [10,11].

**Note S3** Analysis of the utilization of three chilling models in the Auto-ML model

By simply summing the SHAP values of three chilling models and averaging their normalized feature values, their combined effect reveals a standard ‘U’ shape of grapevine LT_50_ across a dormant season (Figure S5b). In the early season when the combined chilling unit accumulation is low, it negatively impacts freezing tolerance (positive SHAP). As the combined chilling unit increases, the negative impact gradually decreases, transitions to a positive impact, and remains relatively unchanged after reaching a turning point (Figure S5b). The turning point (derivative of the loess fitted function $f^{'}\left( SHAP \right)=0$), occurs at 44% of average normalized feature values, corresponding to 1,245 chilling units in averaged Utah, NC, and CU models (Figure S5c). This value coincides with the amount of chilling needed to fulfill chilling requirements for most grapevine cultivars [12].

**Note S4** Daily temperature descriptors and cumulative temperature descriptors

Daily temperature descriptors for the modeling of grapevine freezing tolerance in this study include daily maximum temperature, daily minimum temperature, daily mean temperature and within-day temperature range. Cumulative temperature descriptors include chilling units as estimated from different models and growing degree hours (GDH) with different base temperatures to account for the accumulation of chill and heat throughout the season. Chilling units were estimated with three chilling models, the Chilling Hours model (CU), the Utah model (Utah), and the North Carolina model (NC), using September 1^st^ in each year as the start of dormant season [13–16]. GDHs were computed with the base temperature at 10 °C, 7 °C, 4 °C and 0 °C, starting from January 1^st^ in each year, as a standard approach for the modeling of phenology in perennial plants [17–19]. Cumulative temperature descriptors were computed using R packages ‘chillR’ and ‘fruclimadapt’ [20,21].

**Note S5** Reverse exponential weighted moving average temperatures in the modeling of grapevine freezing tolerance

REWMA temperature is developed in this study to better correlate the physiological response of temperature with grapevine freezing tolerance by formulizing a theory of ‘cold priming’ and the ‘cold shock effect’ through modified EWMA. The cold priming theory is founded on the observations that plants that have been cold-primed and then allowed to recover at warmer temperatures are able to gain freezing tolerance more quickly to cold stress than the plants that have not undergone the initial cold exposure [22–26]. Cold priming was first reported in annual plants such as *Arabidopsis thaliana* and *Brachipodium distachyon* [27,28]. Factoring cold priming in the modeling of *Citrus sinensis* freezing tolerance also resulted in a high accuracy dynamic prediction model [29]. Assuming that cold priming also exists in grapevine, the freezing tolerance in grapevine on a date might not only be modulated by the continuous exposure to a temperature window that are closed to the date (which is addressed using EWMA) but also be impacted by earlier temperature deviants, which might have induced cold priming. Moreover, in grapevine dormant season physiology, earlier condition might sometimes have more impact than current condition, especially during the early stages of cold acclimation when buds are endodormant. A cold shock in early season would significantly enhance the freezing tolerance of endodormant grapevine, and this enhancement would not fade even under a following period of higher temperature. The phenomena is partially explained by the cold acclimation and deacclimation dynamics during accumulation of chilling units [2,10]. However, since we aim to develop a temperature-based prediction model rather than a biology-based prediction model, we should include sufficient thermal features for Auto-ML to factor these theories so that it might identify the underlying biology. To show the potential effect of early sudden temperature abnormality on current freezing tolerance, we exponentially added more weight to earlier temperatures when computing moving average. Compared to the computation of EWMA, the assignment of weight is reversed, thus this method is named REWMA (reverse EWMA).

References:

1. Ferguson JC, Moyer MM, Mills LJ *et al.* Modeling Dormant Bud Cold Hardiness and Budbreak in Twenty-Three Vitis Genotypes Reveals Variation by Region of Origin. *American Journal of Enology and Viticulture* 2014;**65**:59–71.

2. Kovaleski AP, North MG, Martinson TE *et al.* Development of a new cold hardiness prediction model for grapevine using phased integration of acclimation and deacclimation responses. *Agricultural and Forest Meteorology* 2023;**331**:109324.

3. Saxena A, Pesantez-Cabrera P, Ballapragada R *et al.* Grape Cold Hardiness Prediction via Multi-Task Learning. 2022, DOI: 10.48550/arXiv.2209.10585.

4. Soltis PS, Nelson G, Zare A *et al.* Plants meet machines: Prospects in machine learning for plant biology. *Appl Plant Sci* 2020;**8**:e11371.

5. van Dijk ADJ, Kootstra G, Kruijer W *et al.* Machine learning in plant science and plant breeding. *iScience* 2021;**24**:101890.

6. Gall GEC, Pereira TD, Jordan A *et al.* Fast estimation of plant growth dynamics using deep neural networks. *Plant Methods* 2022;**18**:21.

7. Cartwright SJ, Bowgen KM, Collop C *et al.* Communicating complex ecological models to non-scientist end users. *Ecological Modelling* 2016;**338**:51–9.

8. Ellis JL, Jacobs M, Dijkstra J *et al.* Review: Synergy between mechanistic modelling and data-driven models for modern animal production systems in the era of big data. *Animal* 2020;**14**:s223–37.

9. Janiesch C, Zschech P, Heinrich K. Machine learning and deep learning. *Electron Markets* 2021;**31**:685–95.

10. Kovaleski AP, Reisch BI, Londo JP. Deacclimation kinetics as a quantitative phenotype for delineating the dormancy transition and thermal efficiency for budbreak in Vitis species. *AoB PLANTS* 2018;**10**.

11. North M, Workmaster BA, Atucha A. Effects of chill unit accumulation and temperature on woody plant deacclimation kinetics. *Physiologia Plantarum* 2022;**174**:e13717.

12. Londo JP, Johnson LM. Variation in the chilling requirement and budburst rate of wild Vitis species. *Environmental and Experimental Botany* 2014;**106**:138–47.

13. Weinberger JH. Chilling requirements of peach varieties. *Proceedings American Society for Horticultural Science* 1950;**56**:122–8.

14. Shaltout AD, Unrath CR. Rest Completion Prediction Model for ‘Starkrimson Delicious’ Apples. *Journal of the American Society for Horticultural Science* 1983;**108**:957–61.

15. Linvill DE. Calculating Chilling Hours and Chill Units from Daily Maximum and Minimum Temperature Observations. *HortScience* 1990;**25**:14–6.

16. Camargo-Alvarez H, Salazar-Gutiérrez M, Keller M *et al.* Modeling the effect of temperature on bud dormancy of grapevines. *Agricultural and Forest Meteorology* 2020;**280**:107782.

17. Diekmann M. Relationship between flowering phenology of perennial herbs and meteorological data in deciduous forests of Sweden. *Can J Bot* 1996;**74**:528–37.

18. Fu Y, Zhang H, Dong W *et al.* Comparison of Phenology Models for Predicting the Onset of Growing Season over the Northern Hemisphere. *PLoS One* 2014;**9**:e109544.

19. Zapata D, Salazar-Gutierrez M, Chaves B *et al.* Predicting Key Phenological Stages for 17 Grapevine Cultivars (Vitis vinifera L.). *Am J Enol Vitic* 2017;**68**:60–72.

20. Luedeling E, Fernandez E. chillR: Statistical Methods for Phenology Analysis in Temperate Fruit Trees. 2022.

21. Miranda C. fruclimadapt: Evaluation Tools for Assessing Climate Adaptation of Fruit Tree Species. 2023.

22. Schwachtje J, Whitcomb SJ, Firmino AAP *et al.* Induced, Imprinted, and Primed Responses to Changing Environments: Does Metabolism Store and Process Information? *Frontiers in Plant Science* 2019;**10**.

23. Leuendorf JE, Frank M, Schmülling T. Acclimation, priming and memory in the response of Arabidopsis thaliana seedlings to cold stress. *Sci Rep* 2020;**10**:689.

24. Sharma M, Kumar P, Verma V *et al.* Understanding plant stress memory response for abiotic stress resilience: Molecular insights and prospects. *Plant Physiology and Biochemistry* 2022;**179**:10–24.

25. Liu H, Able AJ, Able JA. Priming crops for the future: rewiring stress memory. *Trends in Plant Science* 2022;**27**:699–716.

26. Babajamali A, Gholami M, Baninasab B. Drought preconditioning improves freezing tolerance in drought-tolerant and -intolerant grape cultivars. *Theor Exp Plant Physiol* 2022;**34**:395–407.

27. Zuther E, Schaarschmidt S, Fischer A *et al.* Molecular signatures associated with increased freezing tolerance due to low temperature memory in Arabidopsis. *Plant, Cell & Environment* 2019;**42**:854–73.

28. Mayer BF, Charron J-B. Transcriptional memories mediate the plasticity of cold stress responses to enable morphological acclimation in Brachypodium distachyon. *New Phytologist* 2021;**229**:1615–34.

29. Kimura K, Yasutake D, Oki T *et al.* Dynamic modelling of cold-hardiness in tea buds by imitating past temperature memory. *Annals of Botany* 2021;**127**:317–26.
